# Er:YAG laser ablation: Possibly a valuable tool in the management of refractory Hailey–Hailey disease

**DOI:** 10.1111/jdv.20651

**Published:** 2025-04-25

**Authors:** Danielle Rogner

**Affiliations:** ^1^ Department of Dermatology and Allergology Technical University of Munich, School of Medicine Munich Germany

Management of Hailey–Hailey disease (HHD) remains challenging, traditionally involving symptomatic treatments aimed at controlling flare‐ups and reducing discomfort. However, these treatments can sometimes offer only temporary relief, and they carry risks of side effects.^1^

In order to improve patients' quality of life (QoL), there is a continuing interest in other treatment modalities for HHD. In 1987, Don et al. published the first case report of successful Carbon dioxide laser abrasion in a patient, and since then, there have been various case reports and studies on HHD treated with ablative lasers such as Er:YAG and CO_2_ lasers, indicating that this seems to be a safe and effective therapy.^2^, ^3^

However, there was a lack of in‐depth analysis of this treatment in a large cohort, making the argumentation for the cost‐coverage of this off‐label therapy towards the health insurance difficult, as well as lacking clarification for the exact laser treatment protocol.

The study by Debeuf et al. offers compelling evidence for the efficacy of Er:YAG ablative laser therapy in achieving long‐term remission of HHD and improving QoL. This single‐centre observational study included eight patients with genetically/histopathologically confirmed HHD, and the results showed that a single Er:YAG laser ablation led to complete remission in most of the HHD plaques, even after a median follow‐up of 38 months. It also demonstrated significant improvements in the patients' QoL, measured by Skindex‐29 and DLQI questionnaires.

One of the key strengths is its focus on ultrastructural changes following laser therapy. Electron microscopy revealed that Er:YAG laser treatment restored the number of desmosomes, decreased intercellular distance and diminished perinuclear retraction of keratin filaments in HHD plaques. These findings suggest that laser therapy not only provides clinical improvement but also corrects some of the underlying cellular abnormalities associated with HHD, possibly enhancing the skin's barrier function, therefore also reducing bacterial colonization in these areas and overall decreasing the risk of secondary infections and also viral superinfections.^1^

The findings are consistent with previous studies that have reported successful use of ablative laser therapies in the treatment of HHD. Er:YAG laser ablation is less prone to scarring, and its precision allows for effective targeting of the affected skin areas while minimizing damage to surrounding tissues.^4^

However, the authors also acknowledge the limitations of ablative laser therapy, including the need for accurate and consequent wound care, the potential for scarring and hypopigmentation, and the limitation of treating large body surface areas due to lidocaine dosage limits. Furthermore, they suggest that laser treatment might not be ideal in areas with extensive skin‐on‐skin contact.

Despite these limitations, the study provides strong evidence that Er:YAG ablative laser therapy can be a valuable tool in disease management. While it may not eliminate the disease entirely, it provides a valuable tool in managing flare‐ups and improving the QoL, seemingly leading to long‐term remission. Further research, including larger, multi‐centre, randomized controlled trials, is warranted to confirm these findings and to optimize laser treatment protocols for HHD.^5^

We would like to highlight the importance of initially performing laser therapy on a small skin area to assess the patient's reaction. Our hospital has encountered cases of wound infections, inadequate healing and pain in the treated area.

That said, this research may contribute to achieving cost coverage by health insurance, as laser therapy remains an expensive off‐label treatment for HHD. However, given the significant benefits that some patients experience from a single session, the overall costs could be lower compared to other off‐label treatments.

Managing Hailey–Hailey disease can be challenging for both patients and dermatologists. Therefore, we highly value new insights and treatment options, as we believe every patient deserves access to all possible therapies in the hope of finding one that provides long‐term relief—though, unfortunately, this is not always achievable in HHD.

## CONFLICT OF INTEREST STATEMENT

None to be declared.

## Data Availability

Data sharing not applicable to this article as no datasets were generated or analysed during the current study.
